# Early life stress causes persistent impacts on the microbiome of Atlantic salmon

**DOI:** 10.1016/j.cbd.2021.100888

**Published:** 2021-12

**Authors:** Tamsyn M. Uren Webster, Sofia Consuegra, Carlos Garcia de Leaniz

**Affiliations:** Centre for Sustainable Aquatic Research, College of Science, Swansea University, Swansea, UK

**Keywords:** Microbiota, Temperature, Cold shock, *Salmo salar*, Aquaculture

## Abstract

Farmed fish are commonly exposed to stress in intensive aquaculture systems, often leading to immune impairment and increased susceptibility to disease. As microbial communities associated with the gut and skin are vital to host health and disease resilience, disruption of microbiome integrity could contribute to the adverse consequences of stress exposure. Little is known about how stress affects the fish microbiome, especially during sensitive early life stages when initial colonisation and proliferation of host-associated microbial communities take place. Therefore, we compared the effects of two aquaculture-relevant early-life stressors on the gut and skin microbiome of Atlantic salmon fry (four months post hatching) using 16S rRNA amplicon sequencing. Acute cold stress applied during late embryogenesis had a pronounced, lasting effect on the structure of the skin microbiome, as well as a less consistent effect on the gut microbiome. Follow-up targeted qPCR assays suggested that this is likely due to disruption of the egg shell microbial communities at the initial stages of microbiome colonisation, with persistent effects on community structure. In contrast, chronic post hatching stress altered the structure of the gut microbiome, but not that of the skin. Both types of stress promoted similar Gammaproteobacteria ASVs, particularly within the genera *Acinetobacter* and *Aeromonas*, which include several important opportunistic fish pathogens. Our results demonstrate the sensitivity of the salmon microbiome to environmental stressors during early life, with potential associated health impacts on the host. We also identified common signatures of stress in the salmon microbiome, which may represent useful microbial stress biomarkers.

## Highlights

• The salmon microbiome is sensitive to early life environmental stressors.
• Cold shock just before hatching induced lasting effects on the skin microbiome.
• Pre- and post-hatch stressors also altered the gut microbiome.
• Common stress signatory bacteria including opportunistic fish pathogens

## Graphical abstract

**Figure:**
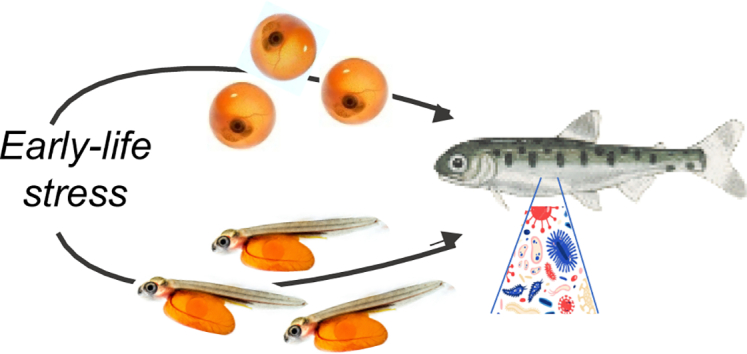

## 1 Introduction

Aquaculture is the fastest growing food producing sector, and plays an increasingly important role in global food security in the face of a growing human population, depletion of capture fisheries and climate change (Teletchea and Fontaine, 2012). However, the intensification of aquaculture is associated with challenges regarding its sustainability, including impact on animal health and welfare, the environment, food safety and economic viability (Stentiford et al., 2017). Intensive aquaculture can expose fish to crowding, handling and social stressors, which can induce adverse effects on health and fitness, such as impaired growth, depressed immunity, and increased susceptibility to disease (Conte, 2004; Ellison et al., 2018; Rodriguez-Barreto et al., 2019). Increasing resistance to stress and disease is therefore a priority in order to improve the sustainability of aquaculture. There is increasing recognition that the microbiome could contribute to this (Perry et al., 2020; Parata et al., 2021).

Host resilience to stress, disease and immune-related disorders is critically influenced by the host-associated microbiome (Rea et al., 2016). In fish, as for other vertebrates, microbial communities in the gut, skin and other mucosal surfaces have a fundamental influence on host health, including nutrient acquisition, metabolism, immune competence and disease resistance (de Bruijn et al., 2017; Koskella et al., 2017; Butt and Volkoff, 2019). In particular, the microbiome plays an important role in the maturation of the vertebrate adaptive immune system and the stimulation of immune response, and can directly enhance host pathogen defence via colonisation resistance and production of inhibitory compounds (Kamada et al., 2013; Kelly and Salinas, 2017). However, the host-microbiota relationship is also sensitive to disruption by environmental stressors (Foster et al., 2017). Stress influences the brain-gut-microbiota axis, including neural, immuno, and endocrine signalling pathways, via complex and interacting mechanisms (Foster et al., 2017). For example, host stress response mediated via the hypothalamus-pituitary-adrenal (HPA) axis (i.e. secretion of corticosteroids and catecholamines) can influence microbiome diversity, structure and function (Huang et al., 2015; Stothart et al., 2016; Uren Webster et al., 2020b), while microbiota and their metabolites also exert modulatory effects on the HPA system, altering stress response (Simard et al., 2014; de Weerth, 2017). Disruption of the gut-brain axis, including microbiome dysbiosis, is linked to adverse health effects in mammals (e.g. Rea et al., 2016; Foster et al., 2017), and is likely to affect fish health and welfare too.

Early life stages are particularly sensitive to environmental stressors, due to developmental plasticity during critical periods for the assembly of the vertebrate microbiome, as well as maturation of the nervous and immune system (Giatsis et al., 2014; Cao-Lei et al., 2017). In mammals, stress during early life has a critical influence on gut microbial colonisation and community establishment, with long lasting effects on both the microbiome and health of the host (Foster et al., 2017); however the sensitivity of the fish microbiome to early-life stress is unknown. The teleost intestine is colonised upon hatching from microbes present in the surrounding water and attached to egg-shell fragments, and these early communities are very dynamic and readily influenced by environmental variation (Giatsis et al., 2014; Ingerslev et al., 2014). Although diet becomes a dominant factor shaping further proliferation and differentiation of the gut microbiota (Giatsis et al., 2014; Smith et al., 2015), historical colonisation effects, reflecting earlier environmental conditions, may have lasting effects on future microbial colonisation (Walter and Ley, 2011; Sprockett et al., 2018; Uren Webster et al., 2020a). This highlights how early life conditions experienced in aquaculture, or natural systems, could have an important, lasting, influence on the fish microbiome and, thus, wider animal health. While stressors disrupting initial microbial community assembly may adversely affect microbiome integrity, early-life microbiome conditioning could also potentially be used to promote host health. However, relatively little is known about the impacts of different types of environmental stress on the fish microbiome, especially during sensitive early life stages.

We hypothesised that early life stress would induce lasting effects on the gut and skin microbiome. To test this, we examined the effects of two contrasting, aquaculture-relevant stressors on the gut and skin microbiome of Atlantic salmon (*Salmo salar*): an acute stressor consisting of cold shock and air exposure during late embryogenesis, and chronic stress induced by lack of tank substrate/shelter for developing fry. These stressors were selected to target pre- and post-hatching developmental stages and were based on existing literature (Hansen, 1985; Moghadam et al., 2017), demonstrating that they induce sub-lethal effects on growth and the immune system without compromising survival. Previously, we documented that these two stressors differentially altered gill transcriptional response to a subsequent pathogenic challenge (Uren Webster et al., 2018). In this study, we characterised the skin and gut microbiome of salmon exposed to each stressor alongside a control (non-stressed) group at four months post hatch using 16S rRNA amplicon sequencing. In order to examine whether the differences observed were due to the disruption of initial microbiome colonisation, we then used a targeted qPCR approach to assess the relative abundance of specific genera in early stage (pre-feeding) post-hatch larvae.

## 2 Materials and methods

### 2.1 Ethics

All experiments were performed with the approval of the Swansea Animal Welfare and Ethical Review Body (AWERB; approval number IP-1415-6), conforming to UK legislation under the Animals (Scientific Procedures) Act 1986.

### 2.2 Fish maintenance

Atlantic salmon eggs from 10 families (obtained from Landcatch Natural Selection, Scotland) were maintained in six vertical incubators (each containing 500 eggs), supplied with flow-through de-chlorinated tap water. Hatched larvae were transferred to six shallow troughs (100L × 40W × 8D) receiving a constant flow-through aerated and filtered water in a recirculating system. Troughs were supplied with artificial substrate (Astroturf) to provide support for egg sac reabsorption and shelter for alevins until emergence. Fry were fed with a commercial salmonid feed (Nutraplus, Skretting, UK) of the appropriate grade and quantity recommended by the manufacturer from 850 degree days (DD). Water oxygen saturation (>90%), ammonia (<0.02 mg/L), nitrite (<0.01 mg/L), nitrate (<15 mg/L) and pH (7.5 ± 0.2) were maintained within appropriate ranges. Water temperature was gradually increased from 9 °C to 11 °C and photoperiod adjusted from 10L:14D to 14L:10D over the four months of the experiments, reflecting seasonal change.

### 2.3 Stress experiments

Eggs were randomly assigned to three experimental groups: non-stressed control, acute cold stress and chronic environmental stress. Each group was maintained in two replicate egg trays/fry troughs as described above, each containing 500 individuals. The acute stress consisted of a cold shock (five minutes immersion in iced water (0.2 °C), followed by five minutes air exposure (12 °C)), during late embryogenesis just prior to hatching (360 degree days; DD). For the chronic stress, hatched larvae were maintained in bare fry troughs lacking the artificial substrate provided to supply support during yolk sac reabsorption and shelter for larvae/fry in the other experimental groups throughout the duration of the experiment (from 475 to 1532 DD). Daily mortalities of embryos, larvae and fry, together with hatching success were recorded. Size (weight & length) was monitored at four time points throughout the experiment (492, 748, 1019 and 1323 DD) based on a subset of 20 euthanised individuals from each replicate tank (40 per treatment), which were then stored in RNA later. At the final sampling point (1532 DD), mass and fork length of 20 fish from each tank were determined and used to calculate Fulton's condition factor.

### 2.4 16S rRNA gene sequencing & bioinformatics

At the end of the four month experimental period (1532 DD) fish were euthanised via an overdose of anaesthetic (Phenoxyethanol; 0.5 mg/L), followed by destruction of the brain according to UK Home Office regulations. Skin mucus was collected using Epicentre Catch-All™ Sample Collection Swabs (Cambio, Cambridge, UK), by swabbing each fish along the entire length of the lateral line five times in both directions, on the left-hand side of the body. Sterile dissection of the whole intestine was performed to include both the intestinal contents and epithelial associated microbial communities. Skin swabs, intestine samples and 50 mL water samples from each tank were stored in sterile tubes at −80 °C until DNA extraction.

16S rRNA gene amplicon sequencing was performed for a total of 10 individuals (five per replicate tank) from each of the three experimental groups (acute stress, chronic stress, control). DNA extraction from the intestine, skin swabs and water samples was performed using a PowerSoil DNA Isolation Kit (Qiagen, Manchester, UK) according to the manufacturer's instructions. Total DNA was quantified using the Qubit HS dsDNA flurometric assay (ThermoFisher, UK). 16S library preparation using Nextera XT Index kit was performed according the Illumina Metagenomic Sequencing Library Preparation guide, amplifying the V4 hypervariable region of the bacterial 16S rRNA gene, then high-throughput sequencing was performed on an Illumina MiSeq platform as fully described previously (Uren Webster et al., 2020a). The primers 519F (5′-AGCMGCCGCGGTAA-3′) and 785R (5′-ACNVGGGTATCTAATCC-3′) were used for skin and water samples but were associated with excessive non-specific amplification of host mitochondrial DNA (12S region) from the gut, therefore the primers 341F (5′-CCTACGGGNGGCWGCAG-3′) and 785R (5′-GACTACHVGGGTATCTAATCC-3′) were used for the gut samples instead.

Analyses of gut, skin, and water samples were performed separately, within Qiime2 (v2019.4, (Bolyen et al., 2019)). Raw sequence reads were initially quality screened and truncated to 280 bp (forward reads) and 240 bp (reverse reads), and the first 8 bp were removed to eliminate potential adaptor contamination. Trimmed reads were then de-noised, merged, subject to chimera screening and removal, and assigned into actual sequence variants (ASVs) using DADA2 (Callahan et al. 2016). Taxonomic classification of ASVs was performed using the Silva reference taxonomy (v132; (Quast et al., 2012)) with a custom trained classifier (Bokulich et al., 2018) specific to the primer pair employed. Mitochondrial sequences (2.91% gut reads and 0.84% skin reads) and chloroplast sequences (4.92% gut reads and 1.64% skin reads) were removed from the dataset, and target reads were subsampled to an equal depth (skin- 6718; gut- 2716; water-8927). Rarefaction curves are included in the supporting information (Fig. S1), and at this subsampling depth all samples had a Good's coverage value of >0.988 indicating good representation of community diversity and structure. Alpha diversity metrics (Chao1 richness, Shannon diversity, Faith's phylogenetic diversity) and beta diversity (Bray-Curtis dissimilarity) were then calculated for all samples. One gut sample (control) and one skin sample (chronic stress) were eliminated from the analysis due to very low read numbers. Negative controls (extraction blanks) prepared and sequenced alongside the samples had very low read numbers (14, 32, 17, 58).

### 2.5 Bacterial relative abundance

Quantitative PCR (qPCR) was used to compare the relative abundance of total bacteria in the gut and skin samples based on 16S gene copy number, using universal bacterial primers (Forward: 5′ TCCTACGGGAGGCAGCAGT, Reverse: 5′ GGACTACCAGGGTATCTAATCCTGTT (Nadkarni et al., 2002). qPCR reactions were performed in a total volume of 10 μL containing 0.25 mM primers with 5 μL iTaq SYBR Green supermix and 1 μL total DNA (diluted 25× in PCR grade water). The reaction conditions consisted of an initial 10 min denaturation step at 95 °C, followed by 40 cycles of 95 °C for 15 s and 59.5 °C for 30s. All samples were run in duplicate, and those from the same tissue were run on the same plate alongside negative controls (water) and positive controls (*E. coli* DNA). Relative bacterial abundance was calculated based on mean Ct values (1/Ct) normalised by total (host and microbial) DNA concentration measured using the Qubit.

Based on the results of the 16S rRNA amplicon sequencing, we also used qPCR to compare the relative abundance of total bacteria, and two target genera (*Acinetobacter* and *Aeromonas*), in whole salmon larvae at an earlier stage of the experiment (748 DD). At this developmental stage, ~five weeks after hatching and just prior to the onset of feeding, salmon larvae have microbial communities associated with their mucosal surfaces, including the skin and the gut, that are distinct from that of the surrounding water (Lokesh et al., 2019). DNA was extracted from 10 whole larvae per group (five per replicate tank) using the DNeasy PowerSoil kit and quantified using the Qubit as before. Alongside relative total bacterial abundance (1/Ct normalised by total DNA concentration), we used genus-specific primers to quantify the relative abundance of *Acinetobacter* (5′ GGYTTACCAAGRCTATACTCAAC and 5′ TACTCATATACCGAAAAGAAACGG; (Bouvresse et al., 2011)) and *Aeromonas* (5′ GCTGTGTCCTTGAGACGTGGC and 5′ TTCTGATTCCCGAAGGCACTCC; (Vandewalle et al., 2012)). All qPCR reactions were run as before using 25× diluted DNA, and optimised annealing temperatures of 56.5 and 60 °C, respectively. For each assay, PCR efficiency was determined using a standard curve (10 fold dilutions of pooled sample DNA), and was between 90 and 105% in all cases. Relative abundance of *Acinetobacter* and *Aeromonas* were determined using efficiency corrected mean Ct values, normalised against the efficiency corrected mean Ct values for total bacteria, according to the 2−ΔΔCt method (Pfaffl 2001).

### 2.6 Statistical analysis

All statistical analysis was performed in R v3.5.0 (R Development Core Team, R and R Core Team, 2019). We employed linear mixed effect models (using the lme4 package (Bates et al., 2015)) to examine the effects of stress treatment and fish size on measures of alpha diversity in the skin and gut, including tank identity as a random factor. We included fish length as a covariate of size effects because it had a lower coefficient of variation than fish mass (CV = 8% vs 28.2%). In each case we achieved model simplification by performing backward selection using the step and drop1 functions and selected the model with the lowest AIC value. We then refitted the final model using Restricted Maximum Likelihood, or as a linear model when tank identity (random component) did not improve model fit. We examined the effects of stress treatment on alpha diversity in the tank water using linear models. Structural analysis (microbial beta diversity) was based on community distance matrices calculated using the Bray-Curtis dissimilarity index. Non-metric multidimensional scaling ordination was performed using the vegan package in R (Oksanen et al., 2019). To examine the impact of stress treatment, fish size (length) and tank identity on community structure, multivariate statistical analysis of community separation (PERMANOVA) was performed using Adonis in the vegan package using 99,999 permutations, while homogeneity of variance was examined using the Betadisper function.

We examined the effect of stress treatment on the abundance of individual ASVs within the gut and skin microbiomes using DeSeq2 (Love et al., 2014), using rarefied data as recommended for microbiome libraries (Weiss et al., 2017). The DeSeq2 models included independent filtering of low coverage ASVs, employed default settings for outlier detection and moderation of ASV dispersion estimates, and optimised power for identification of differentially abundant ASVs at a threshold of alpha = 0.05. ASVs abundance was considered significantly different at FDR <0.05.

To examine the impacts of stress treatment on the relative abundance of total bacteria, *Aeromonas* and *Acinetobacter* (quantified using qPCR) in the gut, skin and larvae, we used linear mixed effect models or linear models (where tank identity did not improve model fit) as described above.

## 3 Results

### 3.1 Fish survival and growth

As described in Uren Webster et al. (2018), neither the acute cold shock (at 360 DD) or chronic post hatching stressor (475–1532 DD) altered overall survival compared to the control group, and we observed no adverse effects on individual health. Although we initially detected a modest reduction (15%) in the weight of chronically stressed fish (pre-feeding), there was no difference in final size (length, weight) or condition factor at the end of the four month experimental period (S Table 1).

### 3.2 Microbiome alpha and beta diversity (four months post hatch)

**Fig. 1:**
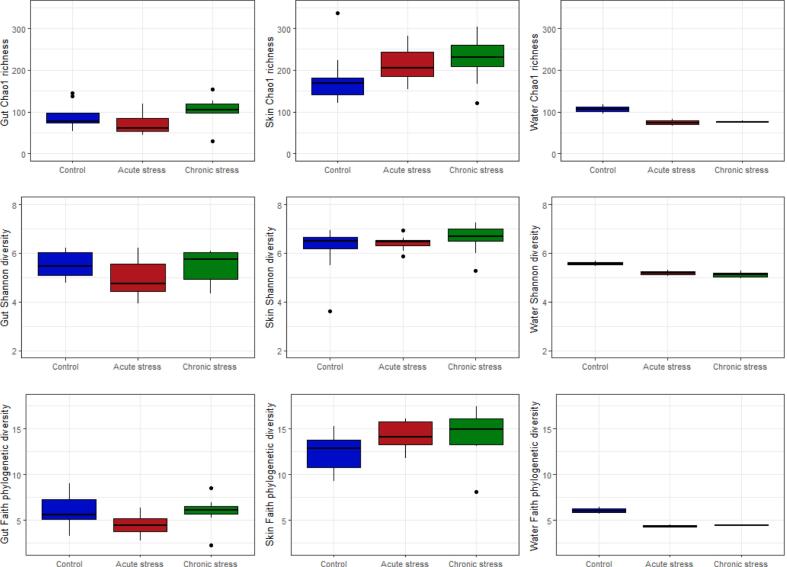
Alpha diversity (Chao1 richness, Shannon diversity and Faith's phylogenetic diversity) in the gut and skin microbiome in fish (n = 10) exposed to an acute cold stress during embryogenesis and a chronic post-hatch environmental stress, and in the tank water from each group (n = 2 replicate tanks) at the time of final sampling (1532 degree days). The control group refers to non-stressed fish maintained in otherwise identical standard hatchery conditions.

At the end of the four month experimental period (1532 DD), using 16S rRNA amplicon sequencing, we found no significant effect of either stressor, or fish size (length), on measures of alpha diversity in the gut or skin microbiome, or in the tank water (Fig. 1). Specifically, for Chao1 richness and Faith's phylogenetic diversity there was no significant effect of either stressor (Chao1- *Gut*: F_2,26_ = 3.11, P = 0.06; *Skin*: F_2,26_ = 1.93, P = 0.17; *Water*: F_2,3_ = 5.54, P = 0.10; Faith- *Gut*: F_2,26_ = 2.86, P = 0.07; *Skin*: F_2,26_ = 3.06, P = 0.06; *Water*: F_2,3_ = 8.74, P = 0.05). For Shannon diversity, there was no detectable effect of either stressor or fish size (P > 0.4 in all cases).

**Fig. 2:**
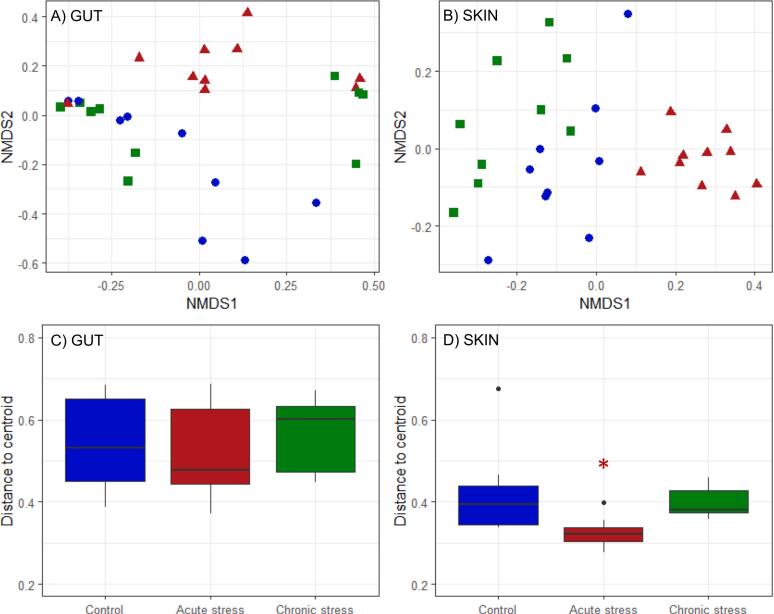
Non-metric multidimensional scaling (NMDS) ordination of microbial gut (A) and skin (B) community structure for individual fish at the final sampling point, based on Bray-Curtis distances (stress scores 0.129 (gut) and 0.177 (skin)) and within-group dispersion in microbial community structure for gut (C) and skin (D). Asterisks indicate significant difference from the control group; * P < 0.05, **P < 0.01, ***P < 0.001.

In contrast, we identified a pronounced effect of early life stress on microbiome structure (beta diversity), based on the Bray-Curtis dissimilarity index, which partitions samples based on community composition (ASV identity and relative abundance) (Fig. 2). Using multivariate analysis of community separation (PERMANOVA) we identified a significant effect of stress treatment, but no effect of fish size (length) or tank identity, on both gut and skin community structure (*Gut*: Stress F_22,2_ = 1.89, P = 0.017, Length F_22,1_ = 0.83, P = 0.605, Tank F_22,3_ = 0.76, P = 0.850; *Skin*: Stress F_22,2_ = 4.01, P = 0.001, Length F_22,1_ = 0.89, P = 0.568, Tank F_22,3_ = 1.21, P = 0.052; Fig. 2a and b). In particular, the skin microbiome of acutely stressed fish was clearly separated from that of the controls and chronically stressed groups. There was also a significantly lower level of within-group dispersion in skin community structure for fish subject to the acute stress, indicating higher similarity in skin community composition for fish in this group, but no significant differences in the degree of gut community structural variance (*Gut*: Stress F_26,2_ = 0.55, P = 0.583; *Skin*: Stress F_26,2_ = 5.42, P = 0.011; Fig. 2c and d). There was no significant effect of stress in the community structure of the tank water samples (F_3,2_ = 2.63, P = 0.133).

We used quantitative PCR (qPCR) to compare the relative abundance of total bacteria in the gut and skin of fish between different stress treatment groups at the final sampling point. For both the gut and skin samples, there was no effect of early life stress on relative total bacterial load (Gut: F_2,27_ = 1.72 P = 0.198, Skin: F_2,27_ = 0.23, P = 0.792).

### 3.3 Microbiome composition and ASV abundance (four months post hatch)

**Fig. 3:**
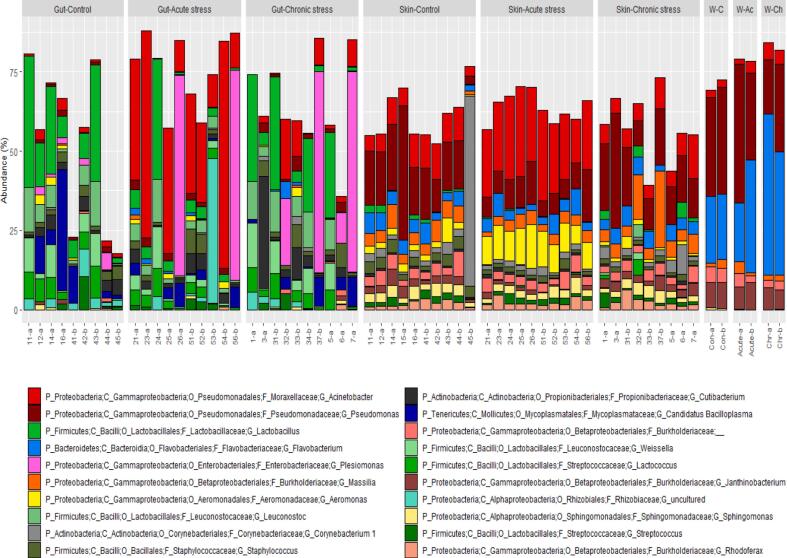
Genus-level composition of the gut, skin and tank water microbiome at the final sampling point, with the samples grouped by treatment in each case. Each bar represents the relative abundance of the top 30 genera, expressed as a percentage of subsampled reads, in one individual fish or water sample. Replicate tank (a/b) and fish identities are indicated in sample name (x axis). Full taxonomy of each genus is included where available (P; phylum, C; class, O; order, F; family; G; genus).

Using 16S rRNA sequencing we found that, overall, the most abundant bacterial phyla present in the gut microbiome were Firmicutes and Proteobacteria, with lower levels of Terenicutes, Actinobacteria and Planctomycetes. The skin microbiome was dominated by Proteobacteria (mainly Gammaproteobacteria), with smaller numbers of Firmicutes, Actinobacteria and Bacteroidetes, while the tank water samples were dominated by Proteobacteria and Bacteroidetes (Fig. 3).

There was a clear effect of both acute cold stress and chronic environmental stress on the composition of the gut microbiome. Using DeSeq2, we identified 65 gut ASVs with significantly different abundance (FDR < 0.05) in acutely stressed fish compared to the controls, and 32 gut ASVs that were differentially abundant between chronically-stressed and control fish. Of these, 24 (75%) were similarly affected by both types of stress (Table S1, Fig. 3). Notably, 25 out of the 36 gut ASVs that were present at higher levels in acutely-stressed fish were members of the class Gammaproteobacteria and, in particular, 19 (53%) were from the genus *Acinetobacter*. Similarly, amongst the 14 gut ASVs present at higher abundance in chronically stressed fish, 11 (79%) were Gammaproteobacteria including five ASVs within the genus *Plesiomonas* and two ASVs within the genus *Acinetobacter*. Overall, *Plesiomonas* and *Acinetobacter* were amongst the most abundant genera in the gut in both stress groups, although there was considerable variation in their abundance between individual fish. Several *Lactobacillus* sp., *Gemmata* sp. and *Candidatus Bacilloplasma* ASVs were amongst those showing the largest decline in abundance in fish subject to both stressors, compared to the controls.

There was also a clear effect of acute cold stress, but not of chronic stress, on the composition of the skin microbiome. We identified 87 individual skin ASVs that were present at significantly different abundance levels in acutely stressed fish compared to control fish, but only one ASV was differentially abundant in fish subject to the chronic stress. Similarly to the gut, a number of Gammaproteobacteria ASVs were present at significantly higher levels in the skin of acutely stressed fish. These included four of the most abundant skin ASVs across all fish; three *Acinetobacter* sp. and one *Aeromonas* sp. (Table S3, Fig. 3). However, in contrast to the gut microbiome, acute cold stress had a far more consistent effect on the skin microbiome between individual fish, also evidenced by lower within-group dispersion in community structure (Fig. 2d).

### 3.4 Relative abundance of selected bacteria in larvae

**Fig. 4:**
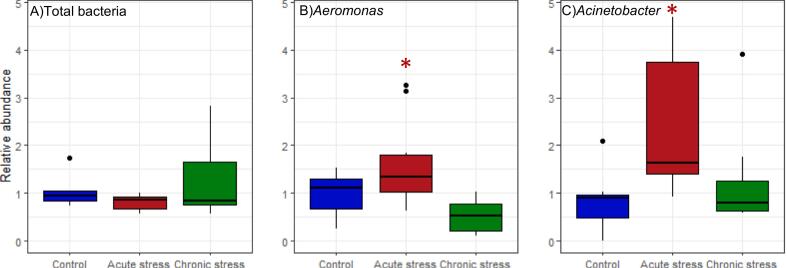
Relative abundance of total bacteria and the target genera *Aeromonas* and *Acinetobacter* in whole salmon larvae between stress treatment groups at an earlier stage of the experiment (748 degree days; 5 weeks post hatch), quantified using qPCR. For visualisation, all values of relative abundance have been normalised by the mean of values in the control group, for that assay. Asterisks indicate significant difference from the control group; * P < 0.05, **P < 0.01, ***P < 0.001.

Using qPCR, we identified no significant effect of early life stress on the relative abundance of total bacteria in larval (pre-feeding) fish (F_2,25_ = 1.88, P = 0.174). However, there was a significant difference in the relative abundance of *Acinetobacter* (F_2,23_ = 4.82, P = 0.018) and *Aeromonas* (F_2,25_ = 7.32, P = 0.003); both genera being elevated in the acutely stressed fish compared to both the control and chronically stressed groups (Fig. 4).

## 4 Discussion

Our results indicate that stress experienced during early life can have persistent effects on the diversity and structure of the Atlantic salmon microbiome. These effects are present even in the absence of effects on fish growth, condition or survival (Uren Webster et al., 2018). Impacts on the microbiome were dependent on the type of stressor, as well as community type (gut or skin), but we also identified some common stress-signatory bacterial taxa. Acute cold stress during late embryogenesis induced very marked and consistent changes in the overall structure of the skin microbiome four months post hatch. This included altered abundance of a large number of individual ASVs and, in particular, a marked increase in *Acinetobacter* sp. and *Aeromonas* sp. In contrast, chronic, post-hatch environmental stress had few discernible effects on skin microbial community diversity or structure. In the gut microbiome, lasting effects of early-life stress were less consistent between individuals, however we observed a general shift from a community largely dominated by Bacilli, especially Lactobacilli, Mollicutes and Planctomycetes, in the control fish, to one dominated by Gammaproteobacteria in both stress groups, with elevated abundance of *Acinetobacter* sp. and/or *Plesiomonas* sp. in particular.

### 4.1 Likely mechanisms of microbiome stress disruption

Microbiome community assembly is determined by complex interactions with the host, the environment and amongst microbiota, which vary with age and body site (Walter and Ley, 2011). While the surrounding water is critical for seeding the initial colonisation of the fish microbiome, host-specific factors, and diet in the case of gut microbiota, tend to become more important with age (Giatsis et al., 2014; Ingerslev et al., 2014). Reflecting this, we found that the gut and skin microbiomes of salmon fry were quite different from that of the tank water. The water microbiome was similar across all tanks and all fish were fed with the same diet in the same way. Together, this indicates that the observed differences in the microbiome of salmon fry were driven by exposure to stressors during early life.

In the case of the acute cold stress during late embryogenesis, we propose that exposure to iced water directly affected the salmon egg shell microbial communities, and thus the subsequent assembly of the gut and skin microbiome. The egg shell microbiome is diverse, strongly influenced by water chemical and physical characteristics, and contributes primary microbial colonisers of the fish intestine and skin upon hatching (Liu et al., 2014; Wilkins et al., 2016). Notably, both *Acinetobacter* sp. and *Aeromonas salmonicida*, are known psychrophiles, with an extensive ability to tolerate and thrive in low temperatures (Doughari et al., 2011; Beaz-Hidalgo and Figueras, 2013). The acute cold shock may therefore have either directly introduced these bacteria to the egg shell microbiome from the iced water and/or favoured these taxa with higher cold tolerance already present in eggshell communities. We found a significant increase in both *Acinetobacter* and *Aeromonas* in very early development (pre-feeding larvae) in the acute cold stress group. Once established, these taxa are likely to have had an enhanced ability to out-compete subsequent colonisers and retain their dominant position through niche pre-emption (Walter and Ley, 2011; Sprockett et al., 2018).

With regard to the chronic stress, we hypothesise that cortisol-mediated stress response may contribute to the observed effects on gut microbiome structure. We found evidence of considerable stress-related transcriptional changes in the gills of these same fish, including genes involved in glucocorticoid production (Uren Webster et al., 2018). A similar chronic environmental stress (lack of tank enrichment) increased cortisol production in juvenile Atlantic salmon (Näslund et al., 2013). In both mammals and fish, elevated glucocorticoid concentrations have been shown to promote Gammaproteobacteria and inhibit probiotic taxa including Lactobacillales (Zijlmans et al., 2015; Stothart et al., 2016; Mudd et al., 2017; Uren Webster et al., 2020b). This is consistent with the observed changes in the gut microbiome of chronically-stressed fish, and the high degree of variation observed between individuals may reflect differences in host stress response i.e. high and low responders in terms of glucocorticoid production (Pottinger and Carrick, 1999).

### 4.2 Potential implications of stress-disruption of the microbiome

While our results suggest that acute cold stress and chronic environmental stress affect the salmon microbiome via different mechanisms, both stressors promoted an increase in the abundance of certain taxa within the class Gammaproteobacteria, especially *Acinetobacter* and *Aeromonas*. The same taxa have previously been linked to hypoxia, and social stress in fish (Ringø et al., 1997; Boutin et al., 2013; Uren Webster et al., 2020b), suggesting they could have an inherently higher resilience to stress, and the ability to thrive in the absence of wider microbial competition (Shade et al., 2012). *Acinetobacter* and *Aeromonas* are both widely distributed in soil, water and as commensals in many animals (Janda and Abbott, 2010; Doughari et al., 2011) but, crucially, also include a number of pathogenic genera that cause significant mortalities and economic loss in aquaculture (Austin and Austin, 2007). Opportunistic *Aeromonas* infections are common in fish subject to stressful conditions (Beaz-Hidalgo and Figueras, 2013), and several *Acinetobacter* species are emergent, opportunistic fish pathogens (Kozińska et al., 2014; Li et al., 2017). Although we did not detect any signs of disease, an increase in the abundance of these pathogenic taxa may enhance risk of opportunistic infection. In the gut we also observed that both acute and chronic stress reduced *Lactobacillus* sp. abundance. Similar reductions have been linked to intestinal inflammation and increased susceptibility to enteric pathogens (He et al., 2013; Liu et al., 2016).

There were no lasting effects on survival or growth of these fish, but in parallel we had found that both of these early life stressors induced considerable transcriptional and epigenetic effects in the gills, in particular linked to immune function (Uren Webster et al., 2018). While it is not possible to directly relate these effects of stress on the gut and skin microbiome with the effects on the immune system identified in the gill, our results highlight how disruption of the microbiome may potentially contribute to broader, interactive effects of stress on Atlantic salmon fry, and should be considered as part of an integrative whole-animal health assessment.

### 4.3 Perspective

Overall, we show that early life stress can induce persistent effects on the microbiome of Atlantic salmon, in a stressor and tissue specific manner, likely through different mechanisms. We demonstrate the sensitivity of the fish microbiome during early development, and the importance of considering the potential impact of subtle, sub-lethal stressors on the microbiome, even in the absence of outward effects on growth and condition. We found that both stressors promoted several important fish pathogens that may increase the risk of opportunistic infections, which is very relevant for intensive aquaculture, where multiple stressors are commonplace. However, as well as demonstrating the potential for disruptive effects, our results highlight how this early life sensitivity could be utilised to condition fish microbiota, promoting the assembly of a robust microbiome that could benefit host health. Furthermore, we identified several bacterial taxa which may represent common microbial stress signatures and could be used as novel biomarkers in aquaculture.

## Funding

This work was funded by a 10.13039/501100000268BBSRC-10.13039/501100000270NERC, United Kingdom Aquaculture grant (BB/M026469/1), the Welsh Government and Higher Education Funding Council for Wales (10.13039/501100000383HEFCW) through the Sêr Cymru National Research Network for Low Carbon Energy and Environment (NRN-LCEE) AQUAWALES project to SC, and the 10.13039/501100008530European Regional Development Fund via WEFO and the SMARTAQUA Operation to CGL.

## Data availability

Sequence reads are available in the European Nucleotide Archive under study accession number **PRJEB32293**; https://www.ebi.ac.uk/ena/data/view/PRJEB32293.

## Declaration of competing interest

The authors declare that they have no known competing financial interests or personal relationships that could have appeared to influence the work reported in this paper.

## Appendix A Supplementary data

Supplementary data: Morphometric data, differentially abundant ASVs, rarefaction curves Image 1
