# Supplementary material for: Early life stress causes persistent impacts on the microbiome of Atlantic salmon
Source: Comp Biochem Physiol Part D Genomics Proteomics. 2021 Dec;40:None. doi: 10.1016/j.cbd.2021.100888 (PMC8600188; doi:10.1016/j.cbd.2021.100888)
Supplement: Supplementary file 1 — Supplementary data: Morphometric data, differentially abundant ASVs, rarefaction curves [file mmc1.pdf]

Table S1. Morphometric data collected throughout the experiment.

	Control A	Control B	Chronic stress A	Chronic stress B	Acute stress A	Acute stress B	Statistical significance
Hatching rate (%)	94.6	96.6	96.6	95.2	94.0	94.6	$F_{3,2}$=1.38, *P*=0.377
Post-hatch mortality rate (%)	11.8	10.8	10.2	11.8	9.2	11.8	$F_{3,2}$=0.19, *P*=0.836
Mass (mg) 492 DD	187.4 ±6.2	185.5 ±10.0	183.3 ±3.5	185.5 ±7.8	183.3 ±6.2	189.6 ±4.6	$F_{3,114}$=0.37, *P*=0.718
Mass (mg) 748 DD	194.3 ±15.2	189.5 ±15.5	156.9 ±13.8	168.6 ±16.6	197.9 ±27.3	189.6 ±19.0	$F_{3,114}$=15.82, *P*=0.025*
Mass (mg) 1019 DD	236.2 ±24.6	246.3 ±37.0	216.7 ±28.3	200.6 ±34.7	249.6 ±34.5	256.6 ±34.8	$F_{3,114}$=15.42, *P*=0.026*
Mass (mg) 1323 DD	287.9 ±43.1	337.0 ±29.8	281.5 ±26.2	262.2 ±27.8	310.7 ±62.6	290.5 ±56.6	$F_{3,114}$=1.63, *P*=0.330
Mass (mg) 1532 DD	381.0 ±73.1	329.8 ±102	348.9 ±76.3	329.9 ±72.5	376.1 ±110	410.7 ±96.1	$F_{3,114}$=4.78, *P*=0.117
Length (cm) 1532 DD	3.54 ±0.19	3.58 ±0.28	3.50 ±0.31	3.39 ±0.26	3.47 ±0.26	3.62 ±0.28	$F_{3,114}$=1.36, *P*=0.381
Condition factor (1532 DD)	0.85 ±0.09	0.84 ±0.09	0.81 ±0.11	0.83 ±0.07	0.86 ±0.07	0.85 ±0.05	$F_{3,114}$=2.18, *P*=0.260

Hatching rate and mortality rate; n=2 replicate tanks/ treatment. Mass and condition factor; n=20 individuals per tank, values presented are mean ±SD. Asterisks indicate significant stress effect (P< 0.05, using linear mixed effect models).

Table S2. Significantly differentially abundant ASVs in the gut

ASV	Mean base abundance	Acute stress log2 fold change	Acute stress FDR	Chronic stress log2 fold change	Chronic stress FDR
G_Acinetobacter	49.92	-4.94	5.91E-12	-1.41	1.18E-01
G_Plesiomonas	46.08	-1.84	3.90E-02	-1.89	4.50E-02
G_Plesiomonas	30.05	-1.79	4.45E-02	-2.08	2.52E-02
G_Plesiomonas	23.10	-2.08	1.48E-02	-2.31	1.09E-02
G_Plesiomonas	18.26	-2.33	4.77E-03	-2.47	6.03E-03
G_Acinetobacter	14.21	-3.96	2.31E-09	-1.39	9.01E-02
G_Acinetobacter	13.32	-3.77	1.06E-08	-1.66	3.38E-02
G_Acinetobacter	13.24	-3.82	6.63E-09	-1.54	5.13E-02
G_Acinetobacter	11.16	-3.47	3.25E-07	-1.63	4.40E-02
G_Gemmata	10.93	3.70	3.25E-07	3.70	1.92E-06
G_ Bacilloplasma	10.15	0.97	2.44E-01	2.27	6.03E-03
G_Acinetobacter	9.97	-3.05	2.97E-05	-1.60	6.34E-02
G_Acinetobacter	9.71	-3.65	6.63E-09	-1.04	2.48E-01
G_Brevinema	9.60	0.28	7.79E-01	1.88	2.52E-02
G_Photobacterium	9.52	2.16	5.28E-03	0.71	5.01E-01
G_Exiguobacterium	9.37	-2.88	5.13E-05	-2.06	1.09E-02
G_Acinetobacter	9.30	-3.13	7.51E-06	-1.60	5.13E-02
G_Acinetobacter	9.30	-3.56	5.59E-08	-0.70	4.73E-01
G_Acinetobacter	8.67	-3.36	6.59E-07	-0.87	3.65E-01
G_Lactobacillus	8.08	2.40	1.65E-03	0.04	9.95E-01
G_Acinetobacter	7.96	-3.26	1.50E-06	-0.78	4.34E-01
G_Hyphomicrobium	7.55	3.33	2.51E-06	3.33	1.21E-05
G_Acinetobacter	7.07	-3.02	1.37E-05	-0.90	3.55E-01
F_Enterobacteriaceae	7.03	-2.02	1.28E-02	-2.07	1.67E-02
G_Gemmata	6.84	3.23	4.62E-06	3.23	1.60E-05
G_Acinetobacter	6.73	-3.09	2.07E-06	-0.91	3.13E-01
G_Acinetobacter	6.73	-3.20	1.00E-06	-0.43	7.03E-01
G_Acinetobacter	6.51	-2.99	7.51E-06	-0.94	3.10E-01
F_Enterobacteriaceae	6.32	-1.43	7.78E-02	-2.08	1.12E-02
G_Acinetobacter	5.88	-2.56	3.17E-04	-1.34	1.28E-01
G_Clostridium	5.59	-0.34	7.19E-01	-1.69	3.38E-02
G_Lactobacillus	5.47	2.77	3.77E-05	1.55	3.53E-02
G_Rhodococcus	5.47	2.44	1.10E-03	0.96	3.10E-01
F_Rhodobacteraceae	5.47	2.97	2.37E-05	2.97	7.88E-05
G_Brevinema	5.36	2.74	1.10E-04	1.88	1.67E-02
G_Reyranella	5.17	2.91	3.15E-05	2.91	9.30E-05
G_Exiguobacterium	4.95	-2.74	5.27E-05	-0.30	7.98E-01
G_Acetobacter	4.87	-2.33	1.79E-03	-1.04	3.04E-01
G_Reyranella	4.72	2.79	6.69E-05	2.79	1.98E-04
G_Acinetobacter	4.43	-2.36	1.16E-03	-0.80	4.34E-01
G_Reyranella	4.28	2.67	1.17E-04	2.67	3.20E-04
1_Proteobacteria	4.20	-2.19	2.52E-03	-1.06	2.75E-01
C_Gammaproteobacteria	4.09	2.62	1.54E-04	2.62	3.83E-04
F_Holosporaceae	3.94	2.58	1.96E-04	2.58	4.51E-04
G_Pseudomonas	3.91	-0.98	2.44E-01	-2.08	1.02E-02
G_Tatumella	3.83	-2.13	3.76E-03	-0.81	4.34E-01
G_ Bacilloplasma	3.65	2.08	4.12E-03	1.01	2.71E-01
G_Acinetobacter	3.46	-2.04	4.77E-03	-0.73	4.73E-01
G_Plesiomonas	3.27	0.02	9.89E-01	-2.17	6.47E-03
G_Flavobacterium	3.16	-2.17	2.04E-03	0.02	9.95E-01
G_Fimbriiglobus	2.97	2.21	1.33E-03	2.21	3.14E-03
G_Lawsonella	2.90	-2.04	4.10E-03	0.02	9.95E-01
G_Photobacterium	2.90	1.50	3.88E-02	0.12	9.67E-01
G_Vibrionimonas	2.83	1.82	9.52E-03	0.96	2.66E-01
G_Streptococcus	2.72	-1.80	1.60E-02	-0.75	4.67E-01
G_Pantoea	2.49	0.02	9.89E-01	-1.82	1.83E-02
G_SH-PL14	2.49	1.52	4.30E-02	0.67	4.73E-01
G_Fimbriiglobus	2.49	1.96	3.76E-03	1.96	7.53E-03

G_Paracoccus	2.42	-1.78	1.38E-02	0.02	9.95E-01
G_Mycoplasma	2.34	1.63	2.27E-02	1.13	1.65E-01
G_Aeromonas	2.34	-1.73	1.55E-02	0.01	9.95E-01
G_Paracoccus	2.34	-1.56	3.35E-02	-0.41	7.07E-01
G_Ulvibacter	2.31	1.67	1.55E-02	1.23	1.10E-01
G_Rothia	2.27	0.01	9.89E-01	-1.68	2.76E-02
G_Pseudomonas	2.16	-1.60	2.48E-02	0.01	9.95E-01
F_Pirellulaceae	2.16	1.70	1.48E-02	1.70	2.36E-02
G_Enterococcus	2.08	1.64	1.81E-02	1.64	2.76E-02
G_Mycoplasma	2.01	1.54	3.80E-02	1.54	4.99E-02
G_Streptococcus	2.01	-1.48	3.88E-02	0.01	9.95E-01
G_Anoxybacillus	1.93	1.54	2.27E-02	1.54	NA
G_Reyranella	1.90	1.44	4.91E-02	1.44	NA
G_Roseococcus	1.82	1.39	4.98E-02	1.39	NA
G_Lactobacillus	1.79	1.37	4.74E-02	1.37	NA

Table S3. Significantly differentially abundant ASVs in the skin

ASV	Mean base abundance	Acute stress log2 fold change	Acute stress FDR	Chronic stress log2 fold change	Chronic stress FDR
G_Pseudomonas	451.15	1.67	0.0016	0.37	0.6138
G_Acinetobacter	267.69	-1.99	8.52E-11	0.64	0.4119
G_Aeromonas	227.05	-1.86	0.0016	0.59	0.4318
G_Acinetobacter	220.11	-2.26	1.09E-04	0.18	0.8735
G_Acinetobacter	173.39	-1.63	4.49E-05	0.84	0.2449
G_Janthinobacterium	157.01	1.27	0.0015	-0.17	0.7840
G_Staphylococcus	145.82	0.88	0.0120	0.98	0.2001
G_Pseudorhodobacter	22.87	-1.99	0.0098	-1.47	0.2976
G_Mycoplasma	18.00	-2.99	3.81E-04	0.99	0.7084
G_Pseudomonas	15.48	-4.47	2.14E-11	-0.04	0.9921
G_Acinetobacter	14.38	-1.89	0.0252	0.51	0.6729
G_Massilia	12.61	3.03	3.81E-04	0.33	0.8048
G_Methylobacterium	10.83	1.93	0.0307	1.06	0.5940
G_Gemmatimonas	10.53	-1.78	0.0374	-2.01	0.2245
G_Massilia	10.49	-1.77	0.0374	-2.81	0.3746
G_Sphingobium	10.35	2.96	3.81E-04	0.84	0.7902
G_Verticia	9.81	-3.62	2.59E-06	-0.70	0.5122
G_Polaromonas	9.28	2.36	0.0066	0.87	0.7911
G_Paracoccus	9.10	-2.15	0.0123	-2.16	0.1834
F_Burkholderiaceae	9.02	-2.92	3.81E-04	-1.97	0.3035
F_Burkholderiaceae	7.91	3.30	7.86E-05	3.27	0.0336
G_Streptococcus	7.58	-2.01	0.0167	1.00	0.6867
G_Rothia	7.55	-1.97	0.0123	-1.74	0.3095
G_Bacillus	7.32	-2.01	0.0233	-1.82	0.2103
G_Streptococcus	7.24	3.24	7.90E-05	3.20	0.0500
G_Hymenobacter	6.91	2.68	0.0010	0.65	0.5124
F_Burkholderiaceae	6.83	-2.27	0.0129	-2.24	0.2144
G_Allorhizobium	6.83	2.64	0.0013	0.65	0.5217
G_Pedobacter	6.80	1.82	0.0374	0.32	0.8071
G_Hyphomicrobium	6.79	-2.05	0.0159	-2.59	0.1447
G_Stenotrophomonas	6.42	2.11	0.0146	1.16	0.4997
G_Acinetobacter	6.33	2.06	0.0168	-0.31	0.8134
G_Chryseobacterium	6.26	-3.10	7.86E-05	-0.46	0.7122
G_Reyranella	6.15	2.32	0.0066	0.22	0.8905
G_Virgibacillus	5.59	2.70	0.0010	1.61	0.2554
F_Burkholderiaceae	5.51	2.00	0.0160	2.70	0.4649
G_Corynebacterium	5.47	-1.98	0.0120	-2.49	0.4352
G_Chryseobacterium	5.28	-2.62	0.0015	-1.05	0.5940

G_Rickettsiella	5.17	-2.86	3.81E-04	-0.03	0.9921
F_Burkholderiaceae	5.13	2.27	0.0062	2.70	0.4352
F_Burkholderiaceae	5.13	-2.45	0.0034	-1.39	0.6105
G_Legionella	4.98	2.47	0.0028	1.17	0.8571
G_Aquabacterium	4.95	-2.49	0.0031	-1.09	0.5819
F_Burkholderiaceae	4.87	-2.77	6.15E-04	-0.03	0.9921
G_Agitococcus	4.87	-1.94	0.0227	-2.03	0.2144
G_Nitrospira	4.42	-2.30	0.0062	-1.23	0.8571
F_Enterobacteriaceae	4.42	-2.72	4.00E-04	-0.02	0.9921
G_Pseudomonas	4.23	-2.62	0.0010	-0.02	0.9921
F_Moraxellaceae	4.15	-1.69	0.0435	-2.00	0.2144
G_Neisseria	3.81	2.26	0.0062	1.47	0.3432
O_Babeliales	3.77	2.01	0.0144	0.66	0.5052
G_Pseudomonas	3.74	-1.84	0.0252	-0.69	0.5122
F_Burkholderiaceae	3.70	-2.08	0.0123	-1.09	0.5493
G_Alkanindiges	3.58	2.43	0.0015	2.41	0.1447
G_Hyphomicrobium	3.55	-1.98	0.0149	-1.20	0.4286
G_Pedobacter	3.32	2.12	0.0079	1.43	0.3296
G_Persicitalea	3.24	-2.08	0.0120	-1.02	0.5977
G_Paludibacter	3.17	-1.88	0.0167	0.25	0.8690
G_Lactobacillus	3.06	2.19	0.0055	2.17	0.1678
G_Clostridium	3.06	2.21	0.0038	2.20	0.1686
G_Acinetobacter	3.02	-1.70	0.0430	-1.11	0.5226
G_Pseudonocardia	2.87	-2.09	0.0080	-0.02	0.9921
G_Hydrogenophaga	2.87	-1.96	0.0123	-0.48	0.6735
G_Mesorhizobium	2.79	1.76	0.0282	0.93	0.5940
F_NS11-12	2.76	-2.04	0.0085	-0.02	0.9921
G_Dolosigranulum	2.76	-1.90	0.0146	0.08	0.9921
O_NB1-j	2.72	-2.01	0.0107	-0.02	0.9921
G_Blastocatella	2.68	1.65	0.0406	0.76	0.8171
G_Schlegelella	2.64	1.99	0.0098	1.97	0.3308
F_Terrimicrobiaceae	2.61	1.93	0.0146	1.91	0.2144
G_Shewanella	2.60	-1.95	0.0120	-0.01	0.9921
G_Alloiococcus	2.57	1.91	0.0149	1.89	0.2225
G_Finegoldia	2.49	-1.67	0.0374	-0.56	0.6107
O_Nostocales	2.45	1.87	0.0136	1.86	0.2144
G_Pseudomonas	2.42	1.76	0.0361	1.74	0.1895
F_Burkholderiaceae	2.41	-1.83	0.0167	-0.01	0.9921
G_Nevskia	2.34	-1.59	0.0443	-0.49	0.6646
F_Verrucomicrobiaceae	2.34	-1.76	0.0233	-0.01	0.9921
G_Flavobacterium	2.34	1.76	0.0233	1.75	0.3558
G_Exiguobacterium	2.30	1.74	0.0252	1.73	0.3808
G_Arenimonas	2.19	-1.66	0.0332	-0.01	0.9921
G_Actinobacillus	2.19	-1.66	0.0332	-0.01	0.9921
G_Chryseobacterium	2.15	1.63	0.0361	1.62	0.1895
F_Burkholderiaceae	2.15	-1.77	0.0233	-0.12	0.9573
G_AAP99	2.08	1.57	0.0423	1.56	0.2157
G_Cereibacter	2.08	1.53	0.0496	1.40	0.3296
G_Perlucidibaca	2.04	-1.55	0.0406	-0.01	0.9921

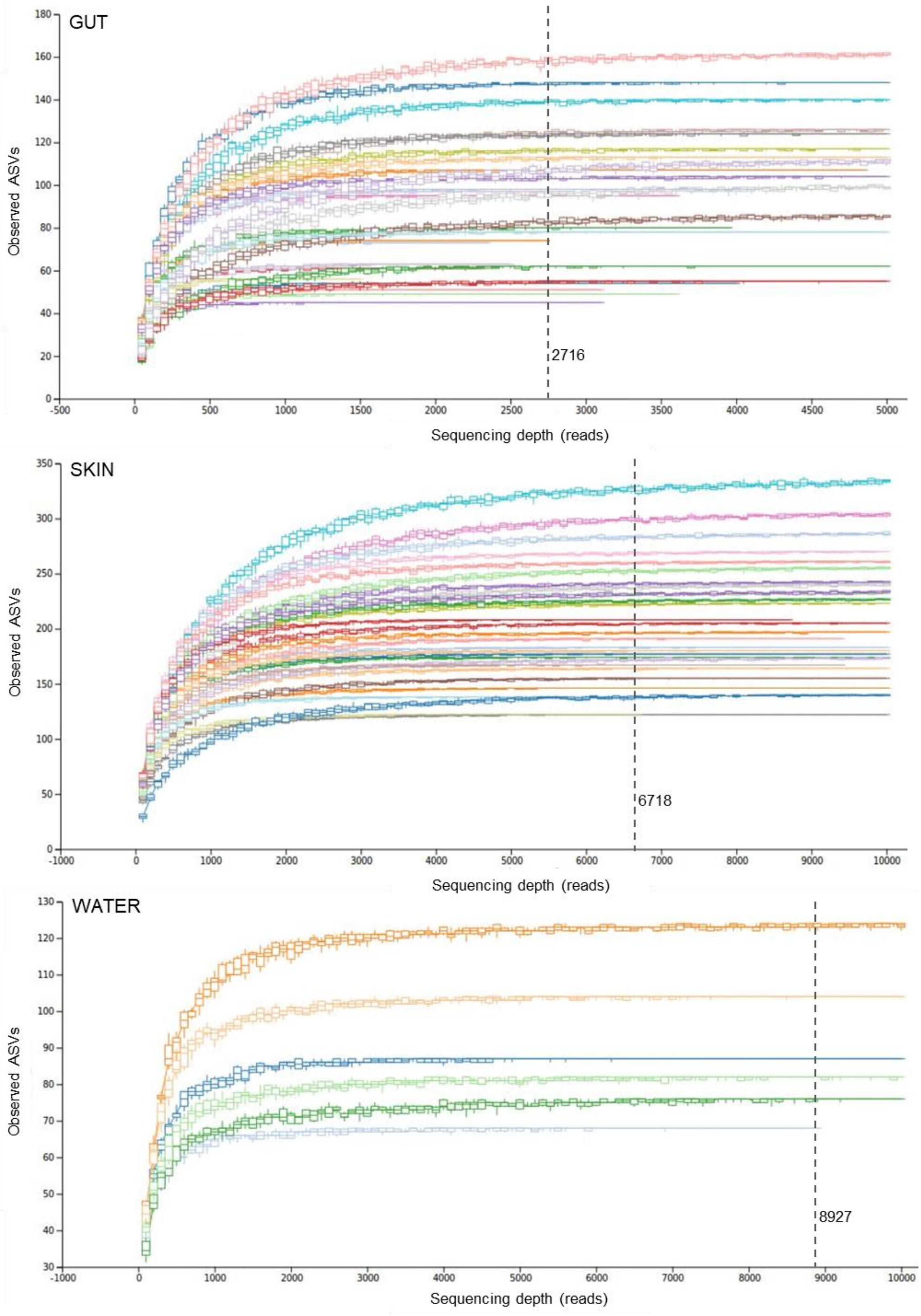

Figure S1. Rarefaction curves for gut, skin and water samples, showing number of observed ASVs at sequential 100 read sampling steps. The minimum read depth, at which subsampling was performed is indicated with a dashed line in each case.
